# Association of estimated glomerular filtration rate with prostate cancer risk in a cross-ethnic population: a Mendelian randomization study

**DOI:** 10.1186/s12894-024-01402-1

**Published:** 2024-01-23

**Authors:** Haojian Zhang, Tian Li, Yingjie Jia

**Affiliations:** 1 Tianjin University of Traditional Chinese Medicine, Tianjin, 300381 China; 2 First Teaching Hospital of Tianjin University of Traditional Chinese Medicine, Tianjin, 300381 China; 3 National Clinical Medical Research Center of Acupuncture and Moxibustion, Tianjin, 300381 China; 4 School of Basic Medicine, Fourth Military Medical University, Xi’an, 710032 China

**Keywords:** Mendelian randomization, Estimated glomerular filtration rate, Prostate cancer

## Abstract

**Objective:**

To investigate whether a causal relationship exists between the estimated glomerular filtration rate (EGFR) and the occurrence of prostate cancer in East Asian and European populations and to determine if genetic factors influence the association between the EGFR and prostate cancer risk.

**Methods:**

In this Mendelian randomization study, the existence of a causal relationship between the EGFR and prostate cancer occurrence was assessed using five analytical techniques, including Mendelian randomization-Egger regression (MR-Egger), calculation of the weighted median estimator (WME), the maximum likelihood ratio method, the linear median weighting method and the random-effects inverse-variance weighting (IVW) method.

**Results:**

In the IVW model, no causal relationship was observed between the EGFR and prostate cancer in either the East Asian or European populations.

**Conclusions:**

After excluding confounding factors and reverse causal associations using two-sample Mendelian randomization, unbiased estimates were obtained, and there was no causal relationship between prostate cancer and the EGFR in the East Asian or European populations. Therefore, for patients with suspected prostate cancer, it is considered unnecessary to improve the detection of glomerular filtration rate, which will effectively reduce the economic burden of patients.

## Introduction

Globally, prostate cancer has increasingly become a high-risk oncological disease; it is the second most common type of cancer in men and was the fifth leading cause of cancer-related deaths in 2020 [1]. The incidence of prostate cancer varies geographically, with the highest incidence observed in Europe and the Caribbean, and Asia and North Africa have the lowest fatality rates related to the disease. Very little is known about the causes of prostate cancer, although certain risk factors have been identified, including age, family history, genetic mutations, and a history of other diseases. Although previous studies have suggested that several modifying factors may increase the risk of prostate cancer, including certain nutritional factors, smoking, and being overweight, they have failed to provide definitive evidence that they are risk factors [2]. The identification of the risk factors prostate cancer is crucial for disease prevention and the development of early screening protocols.

It is possible that renal function could influence the pathophysiology of prostate cancer. Chronic kidney disease is common, with a prevalence of approximately 5–15% in the general population in most developed countries [3, 4].Even minor changes in the glomerular filtration rate can lead to a significantly higher risk of experiencing complications such as cardiovascular disease [5], infections [6], anemia [7], bone fractures [8], and possibly cancer. Renal dysfunction leads to the retention of metabolic waste and the disruption of several signaling pathways associated with cancer, including those involved with the regulation of the immune system [9, 10], inflammation [11], and vascular endothelial cell abnormalities [12]. Several clinical studies have reported a strong correlation between end-stage renal disease and tumor development [13, 14]. However, there is a lack of convincing evidence to confirm whether non-end-stage renal disease leads to an increased incidence of cancer, particularly prostate cancer, which is currently the most prevalent cancer of the urinary tract. Although recent studies have confirmed a higher detection rate of prostate cancer in patients with abnormal renal function than in those with normal renal function [15], some studies have found no significant correlation between the estimated glomerular filtration rate and the incidence of prostate cancer [16]. This discrepancy may be related to differences in the characteristics of the studies and the populations involved. The presence of chronic kidney disease could also be a potential confounding factor, as those with the disease have greater access to cancer screening than those who do not, and observational studies have inferred the presence of a causal relationship between renal dysfunction and prostate cancer when it could in fact be merely correlational in nature.

To clarify whether the EGFR is a risk factor for prostate cancer, this study aimed to determine whether a causal relationship exists between the two variables using Mendelian randomization (MR), which is a powerful analytical method for identifying causal relationships between risk factors and diseases, with genetic variability as an instrumental variable.

## Materials and methods

### Data sources and study design

A genome-wide association study (GWAS) was performed to confirm that the exposure variable was the EGFR and the outcome variable was prostate cancer. Pooled GWAS data for the EGFR and prostate cancer were obtained separately for the European and East Asian populations. The GWAS data for the EGFR in the European population were obtained from the Genome-wide Association Study Consortium database, which contained data from 76,511 samples with a total of 7,892,788 single nucleotide polymorphisms (SNPs). The GWAS EGFR data for the East Asian population were obtained from the Japan Biobank, which contained data from 143,658 samples with 6,593,277 SNPs. For the European population, the GWAS data for prostate cancer were obtained from the Prostate Cancer Association Group to Investigate Cancer Associated Alterations in the Genome (PRACTICAL) consortium database, which contained a total of 140,254 samples(including 79,148 samples in the observation group and 61,106 samples in the control group). The observation group was prostate cancer patients, and the control group was healthy volunteers. In the East Asian population. The GWAS data for prostate cancer were obtained from the Japan Biobank, which contained 109,347 samples, (including 5,408 samples in the observation group and 103,939 samples in the control group), with a total of 8,878,753 SNPs. Specific brief information is shown in Table 1. The existence of a causal relationship between the EGFR and prostate cancer risk was assessed using the designed two-sample MR (TSMR) model.

**Table 1:** Summary of the GWAS DATA included in this two-sample mendelian randomization study

Exposure factors/outcome factors	Data sources	Total sample size	Number of SNPs
GWAS data on the EGFR in European populations	GWAS Consortium for Genome-Wide Association Studies	76,511	7,892,788
GWAS data on the EGFR in East Asian populations	Biobank Japan	143,658	6,593,277
GWAS data on prostate cancer in European populations	PRACTICAL Alliance	140,254	20,346,368
GWAS data on prostate cancer in East Asian populations	Biobank Japan	109,347	8,878,753

### Selection of instrumental variables

Genetically variable SNP loci with genome-wide significance (*P* < 5 × 10^-8^) for the determination of the EGFR were selected for pooling using R software. The linkage disequilibrium parameter (r^2^) was set at a threshold of 0.1, and a genetic distance of 1,000 kb was used to select SNPs; these parameters were selected to ensure independence and to prevent linkage disequilibrium (LD) from influencing the results. The remaining SNPs were retrieved from the Human Genotype-Phenotype Association Database, with a focus on excluding phenotypes associated with SNPs were associated with prostate cancer, as well as those that met the following criteria: (1) a significant association with the exposure variable; (2) no association with the outcome variable; and (3) no association with confounding factors. GWAS information on the outcomes of prostate cancer was extracted, and we combined exposure and outcome datasets. The remaining SNPs represented the final instrumental variables referring to the exposure variable.

### TSMR methods

The TSMR was conducted using the following five methods: (1) MR-Egger regression (MR-Egger); (2) the weighted median estimator (WME) method; (3) random-effects inverse-variance weighting (IVW) modeling; (4) the maximum likelihood ratio method; and (5) the linear median weighting method.

### Sensitivity analysis

Cochran’s Q test was performed using the MR package in R software(version4.2.3) for SNPs that met the three hypotheses in order to assess heterogeneity between individual genetic variants. Such heterogeneity was defined as *P* < 0.05 in Cochran’s Q test, indicating that the relationship between the exposure and outcome variable was influenced by other factors such as age and sex According to the final MR results, the gold standard is the IVW random-effects model; otherwise, as a gold standard, the IVW fixed-effects model was used, and the degree of heterogeneity was depicted visually using forest plots. The MR-Egger intercept test and the MR Pleitropy RESidual Sum and Outlier (MR-PRESSO) test were conducted to test for violations of the MR assumptions due to horizontal multi-effects. For the MR-Egger intercept method, the cut-off value estimates whether a selected instrumental variable (in this case, genetic variability) significantly affects an outcome through alternative pathways that do not involve the exposure variable, with *P* < 0.05 indicating the presence of horizontal pleiotropy. In this method, *P* > 0.05 indicated that the exposure did not significantly affect the outcomes through alternative pathways. The leave-one-out test was used for the sensitivity analysis to determine whether any of the final SNPs were outliers. The stability of the results was verified by examining the asymmetry in the funnel plot. The MR-PRESSO method was subsequently used to identify outliers and assess their impact on the results.

### Evaluation of instrumental variables

The F statistic was calculated to quantify the strength of genetic variability, with F > 10 indicating a strong correlation. All methodological sections were obtained using R4.2.3.

## Results

### Final instrumental variables in the TSMR

The genetically variable SNP loci with genome-wide significance (*P* < 5 × 10^− 8^) for the EGFR were selected for pooling using R software. A threshold of 0.1 was set for the linkage disequilibrium parameter (r^2^), and a genetic distance of 1,000 kb was used. The SNPs that simultaneously satisfied all three principles of MR were screened. Initially, 47 SNPs were extracted from the GWAS data for the EGFR in the European population. Data for 47 SNP-related phenotypes were retrieved based on the human genotype-phenotype association database, excluding SNPs with corresponding phenotypes that were relevant to prostate cancer with significant correlations (*n* = 0). The GWAS information was extracted for prostate cancer as the outcome, the exposure and outcome datasets were combined, and echo SNPs were excluded, including rs10515085, rs187355703, rs62021209, rs6711001, rs72827901, rs744103, rs7735249, rs80282103, and rs8096658, resulting in 38 remaining SNPs. The 38 remaining SNPs were selected as final instrumental variables referring to exposure.

Subsequently, GWAS data on the EGFR in Asian populations were extracted, and 22 SNPs were included. Data for 47 SNP-related phenotypes were retrieved from the human genotype-phenotype association database, excluding SNPs whose corresponding phenotypes were relevant to prostate cancer (*n* = 0). GWAS information on the outcome of prostate cancer was extracted, and the exposure and outcome datasets were combined, with no echo SNPs. The full set of SNPs were used as the final instrumental variables related to the exposure.

### MR analysis

**Fig. 1:**
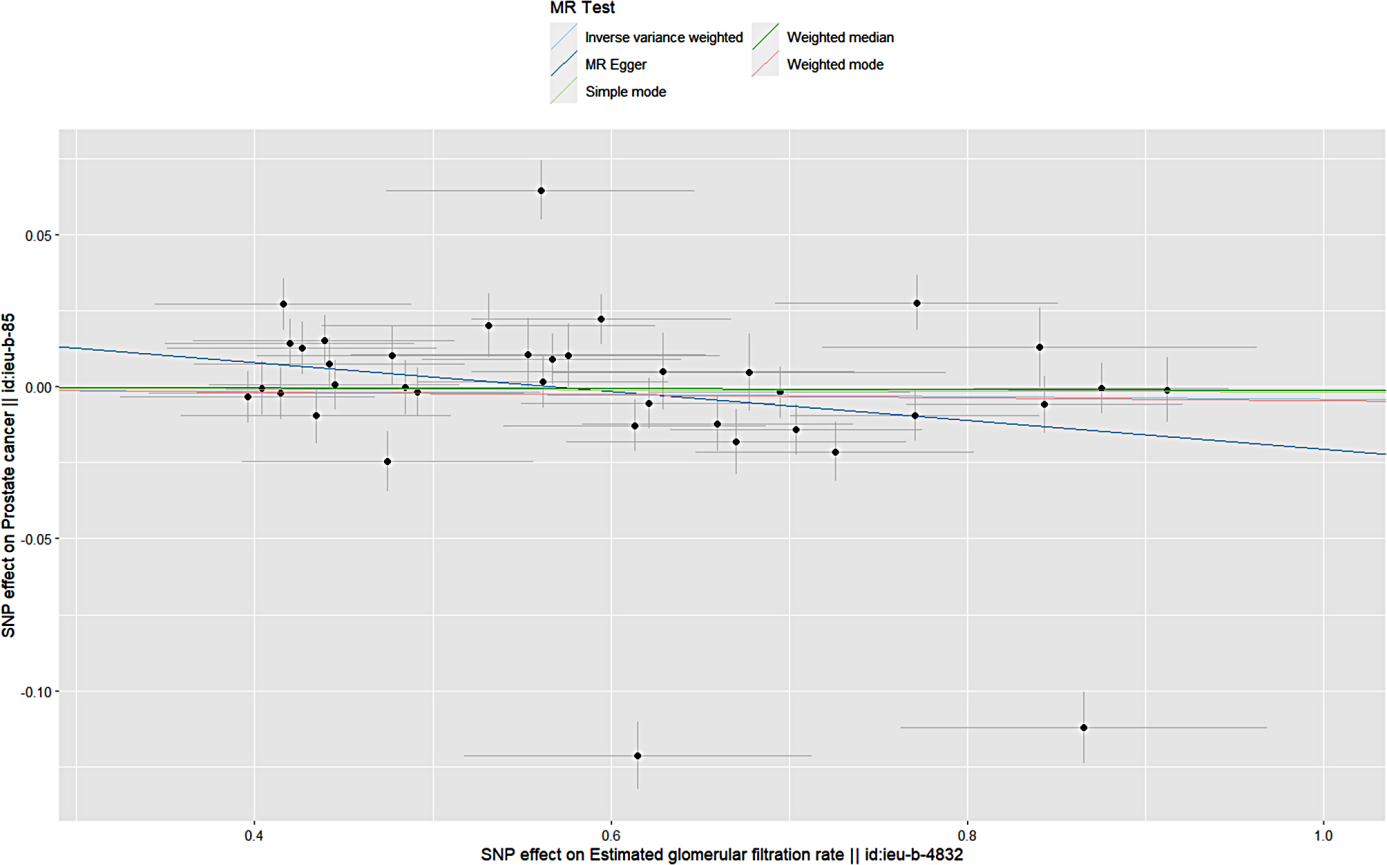
Scatter plot of estimated glomerular filtration rate and prostate cancer in the European population

**Fig. 2:**
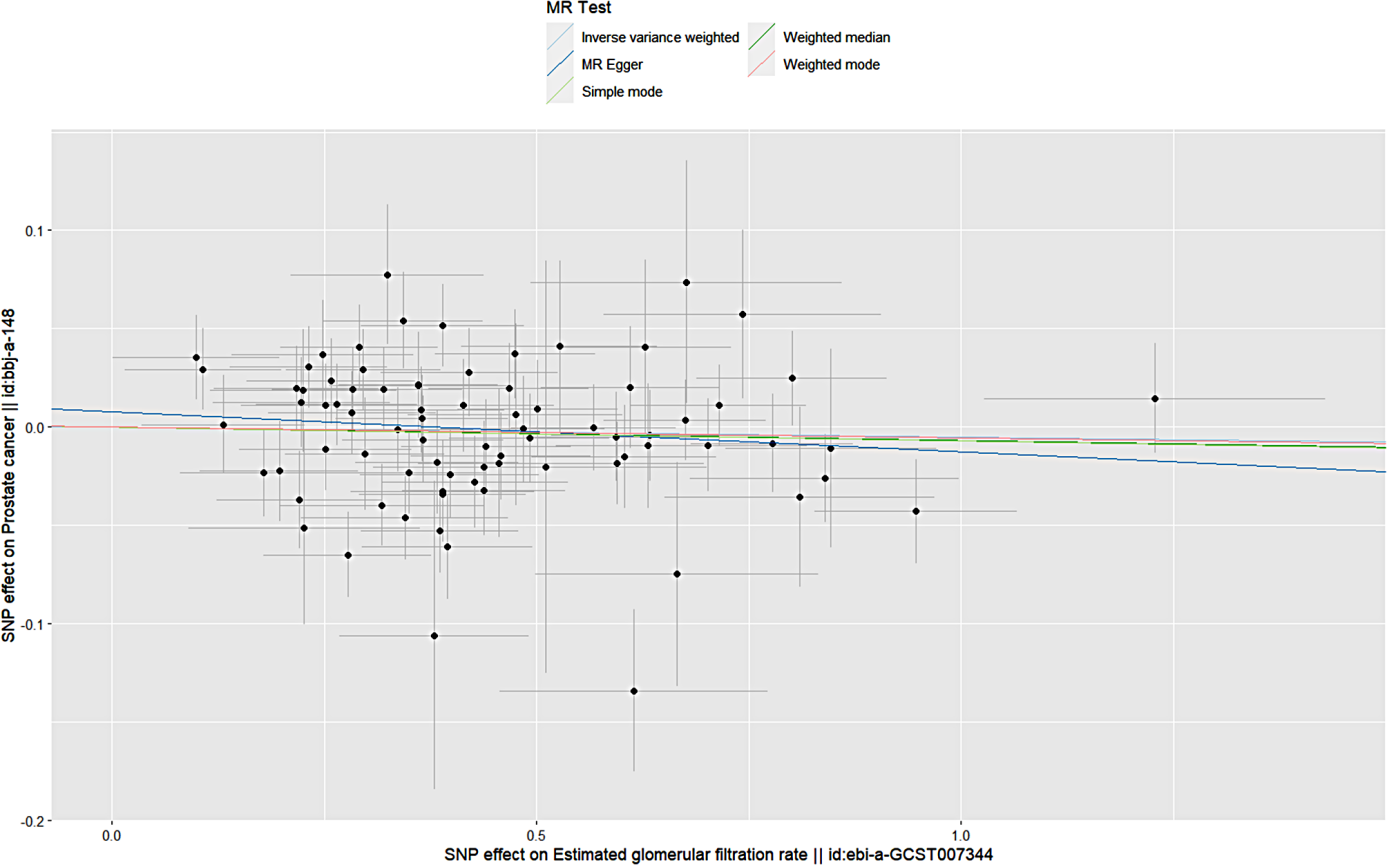
Scatter plot of estimated glomerular filtration rate and prostate cancer in an East Asian population

We have reviewed “Guidelines for Reporting of Statistics for Clinical Research in Urology” to improve the reporting of heterogeneity and diversity in this study [17]. The MR analysis revealed that genetically predicted elevation of the EGFR was not associated with an increased risk of prostate cancer, and the direction of the causal effect was consistent across the five methods. The primary method of analysis, the IVW method, revealed no statistically significant association between an increased EGFR and European men’s prostate cancer risk (odds ratio (OR) = 1.0; 95% confidence interval (CI) = 0.9-1.0;*P* = 0.6). The estimated association between an increased glomerular filtration rate and the risk of prostate cancer in the East Asian population was not statistically significant (OR = 1.0;95% CI = 0.9-1.0;*P* = 0.6). Specific brief information is shown in Figs. 1 and 2.

### One-by-one elimination test

**Fig. 3:**
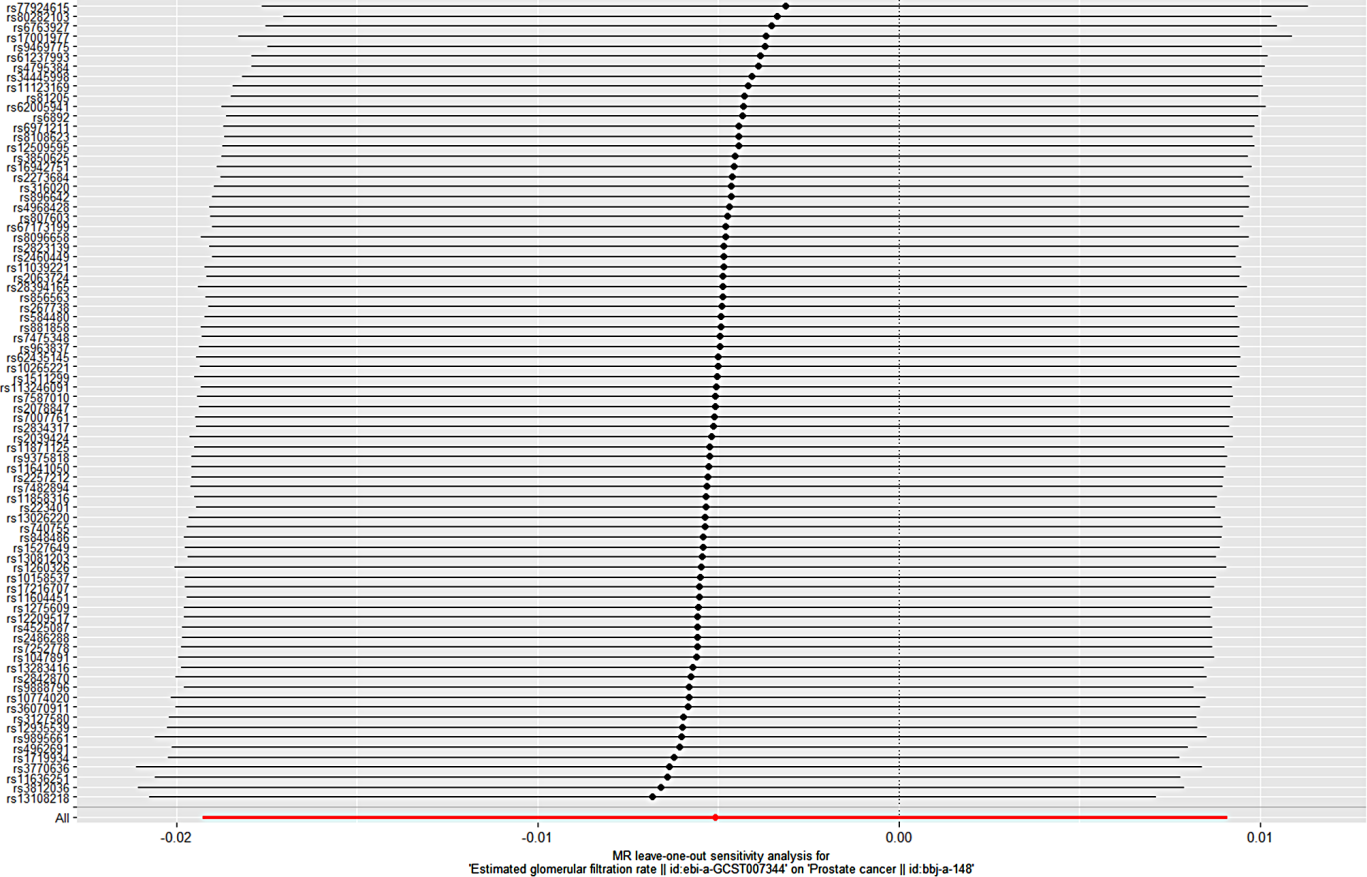
Graph of estimated glomerular filtration rate and prostate cancer retention in European populations

**Fig. 4:**
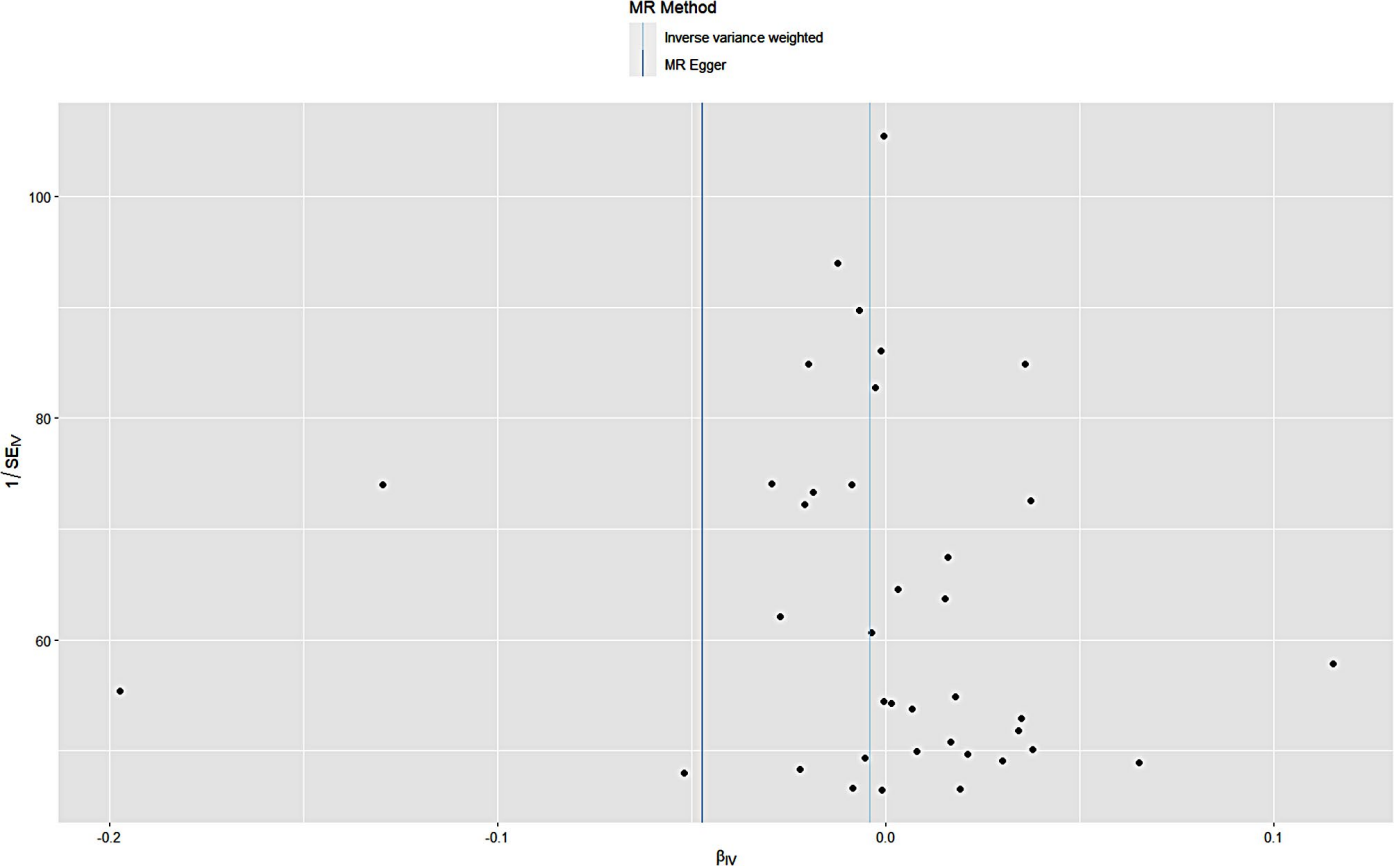
Estimated glomerular filtration rate and prostate cancer funnel plot in European population

**Fig. 5:**
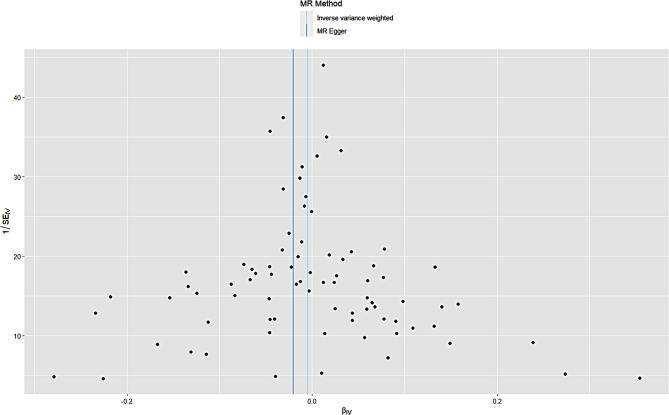
Funnel plot of estimated glomerular filtration rate and prostate cancer in an East Asian population

**Fig. 6:**
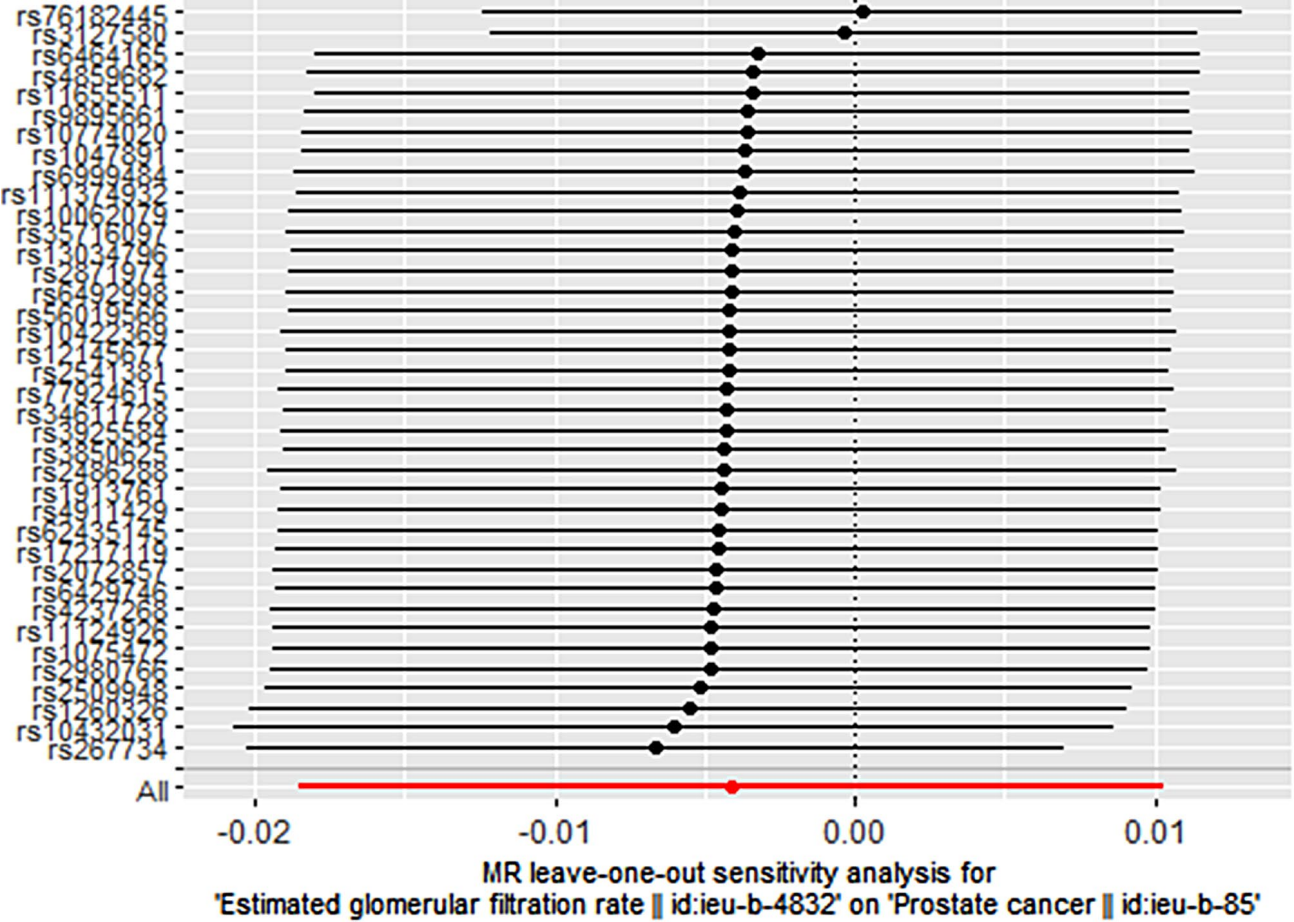
Estimated glomerular filtration rate and prostate cancer retention in East Asian populations

After the “leave-one-out” sensitivity analysis was conducted using the IVW method, No SNPs that had a greater impact on disease outcomes were found. Specific brief information is shown in Figs. 3 and 4. Funnel plot shows that there is no directional pleiotropy. Specific brief information is shown in Figs. 5 and 6.

## Discussion

Neoplasm remains the main killer worldwide [18–21]. There has been some controversy among past studies as to whether glomerular filtration rate is associated with the risk of prostate cancer. Another study that included 3782 patients confirmed that glomerular filtration rate was a negative correlate of the percentage of free prostate-specific antigen, while a percentage of free prostate-specific antigen < 0.16 was a high-risk factor for prostate cancer [22]. Bruun et al. corroborated this view by performing regression analysis and confirmed a significant correlation between glomerular filtration rate and percentage of free prostate-specific antigen [23].

Unlike the above studies, another undifferentiated study denied the association between glomerular filtration rate and the risk of prostate cancer development, Kim et al. confirmed that the percentage of free prostate-specific antigen was not correlated with glomerular filtration rate by correlation and multivariate regression analyses of percentage of free prostate-specific antigen, body mass index, prostate size, and glomerular filtration rate of 91 patients with prostate cancer [24]. Mok et al. quantified the relationship between glomerular filtration rate and cancer risk using a Cox regression model corrected for potential confounders and confirmed that glomerular filtration rate was not significantly associated with prostate cancer [25].

Given that current studies disagree on estimating the relationship between glomerular filtration rate and prostate cancer, the present study, in an effort to further elucidate this issue, used a large GWAS database. A total of 38 SNPs were screened in the European population for associations with both the EGFR and prostate cancer, and five complementary MR methods were used to analyze the causal relationship between the two variables.

There are four main innovations in this study. First, this study used the Two Sample MR package in R software to integrate the screened SNP loci, to exclude SNPs directly related to prostate cancer risk factors, and to fully consider SNPs with a greater impact on disease outcome and eliminate them. Second, using strict quality control conditions and analytical techniques, five complementary MR analysis methods were used to explore the causal relationships between variables, and two different sensitivity analyses were conducted to verify the robustness of the results. Third, the use of MR methods minimized the potential influence of confounding factors and reverse causality on the results. Fourth, the final results based on five Mendelian randomization methods are consistent., with a high degree of feasibility, confirming that there is no causal relationship between the EGFR and prostate cancer.

The study had some limitations. 1.the two-sample MR method assumes that a linear relationship exists between the exposure factor (EGFR) and the disease outcome (prostate cancer), whereas the MR method is not applicable if the relationship is non-linear2.Database statistics are difficult to be stratified by gender or age, which may lead to bias in the results of the study.

In conclusion, the results of this study suggest that there is no causal relationship between estimated glomerular filtration rate and prostate cancer and that estimated glomerular filtration rate is not a risk factor for prostate cancer development, and screening for prostate cancer in a population with an abnormal glomerular filtration rate has limited clinical relevance.

## Data Availability

https://www.jianguoyun.com/p/DdateikQuaiFChj8hZMFIAA.

## Abbreviations

EGFR: Estimated glomerular filtration rate
MR-Egger: Mendelian randomization-Egger regression
WME: Weighted median estimator
IVW: Inverse-variance weighting
MR: Mendelian randomization
GWAS: Genome-wide association study
SNPs: Single nucleotide polymorphisms
PRACTICAL: Prostate Cancer Association Group to Investigate Cancer Associated Alterations in the Genome
TSMR: Two-sample MR
LD: Linkage disequilibrium
MR-PRESSO: MR Pleitropy RESidual Sum and Outlier
OR: Odds ratio
CI: Confidence interval
